# TGF-β1 Drives Inflammatory Th Cell But Not Treg Cell Compartment Upon Allergen Exposure

**DOI:** 10.3389/fimmu.2021.763243

**Published:** 2022-01-07

**Authors:** Stephanie Musiol, Francesca Alessandrini, Constanze A. Jakwerth, Adam M. Chaker, Evelyn Schneider, Ferdinand Guerth, Benjamin Schnautz, Johanna Grosch, Ileana Ghiordanescu, Julia T. Ullmann, Josephine Kau, Mirjam Plaschke, Stefan Haak, Thorsten Buch, Carsten B. Schmidt-Weber, Ulrich M. Zissler

**Affiliations:** ^1^ Center of Allergy & Environment (ZAUM), Technical University of Munich (TUM) and Helmholtz Center Munich, German Research Center for Environmental Health, Members of the German Center of Lung Research (DZL), Munich, Germany; ^2^ Department of Otorhinolaryngology, Klinikum rechts der Isar, TUM School of Medicine, Technical University Munich, Munich, Germany; ^3^ Institute of Laboratory Animal Science, University of Zurich, Zurich, Switzerland

**Keywords:** TGF-beta, Th2, Th9, Th17, asthma, allergen-specific immunotherapy, induced sputum

## Abstract

TGF-β1 is known to have a pro-inflammatory impact by inducing Th9 and Th17 cells, while it also induces anti-inflammatory Treg cells (Tregs). In the context of allergic airway inflammation (AAI) its dual role can be of critical importance in influencing the outcome of the disease. Here we demonstrate that TGF-β is a major player in AAI by driving effector T cells, while Tregs differentiate independently. Induction of experimental AAI and airway hyperreactivity in a mouse model with inducible genetic ablation of the gene encoding for TGFβ-receptor 2 (*Tgfbr2*) on CD4^+^T cells significantly reduced the disease phenotype. Further, it blocked the induction of pro-inflammatory T cell frequencies (Th2, Th9, Th17), but increased Treg cells. To translate these findings into a human clinically relevant context, Th2, Th9 and Treg cells were quantified both locally in induced sputum and systemically in blood of allergic rhinitis and asthma patients with or without allergen-specific immunotherapy (AIT). Natural allergen exposure induced local and systemic Th2, Th9, and reduced Tregs cells, while therapeutic allergen exposure by AIT suppressed Th2 and Th9 cell frequencies along with TGF-β and IL-9 secretion. Altogether, these findings support that neutralization of TGF-β represents a viable therapeutic option in allergy and asthma, not posing the risk of immune dysregulation by impacting Tregs cells.

## Introduction

The three isoforms of transforming growth factor-β (TGF-β), TGF-β1, TGF-β2 and TGF-β3 in mouse and human, encoded by separate genes, are involved in a plethora of biological processes during development, lineage commitment, wound healing, proliferation, migration and survival of cells (1). Each isoform and the TGF-β receptors (TGF-βR) are expressed in specific and temporal patterns making their functions strongly context-dependent for various tissues and cell types (2). TGF-β was first described as suppressor of T cell proliferation (3). Within the immune system TGF-β signaling was later found to have essential roles in the T cell, B cell and phagocyte compartments, among others. Initially it was thought that TGF-β was a checkpoint molecule of T cell-mediated autoimmune inflammatory disease, mediated by Treg dependent (4) and independent (5) mechanisms. However, later it was found that this phenomenon was dependent on a lymphopenic environment (6, 7). TGF-β is now known to be involved, and in some cases essential, for the development of regulatory T (Treg) cells, for differentiation and lineage commitment of Interleukin-17 producing T helper (Th17) cells (mouse only), of follicular T helper (Tfh) cells (human only) and of IL-9 producing T helper (Th9) cells (8, 9). Furthermore, TGF-β is an important inducer of integrins, particularly of CD103 in CD8^+^ resident T memory cells (10), Th1 cells (11), and Treg cells (12, 13), which makes it also a regulator of cell migration during and after inflammation. The various outcomes of TGF-β signaling are achieved through combinatorial sensing of additional cytokines (such as IL-6, IL-1, IL21 in combination with TGF-β for Th17 induction), hence result from specific local cytokine milieus (14).

Allergic diseases are characterized by an uncontrolled immune reaction towards harmless environmental antigens to which the body is exposed either *via* airways, as seen in allergic rhinitis (AR) and allergic asthma (AA), *via* skin (atopic dermatitis), gastrointestinal tract (food allergy) or by systemic exposure (anaphylaxis). In healthy individuals, allergen exposure is tolerated. Loss of T cell tolerance towards environmental antigens is a prerequisite for initiation of an allergic reaction and can lead to activation of Th2 cells, humoral (IgE) effector mechanisms as well as infiltration of inflammatory cells at the site of allergen exposure. At these tissue sites, increased mucus production (15), smooth muscle (16) and airway epithelial cell activation (17) are observed, subsumed as airway hyperreactivity (AHR).

In airway inflammation TGF-β1 is involved in tissue remodeling (18), yet all isoforms are expressed throughout the normal lung, including expression by bronchial epithelium, macrophages, vascular endothelium, smooth muscle and fibroblasts (19, 20). TGF-β is also a core inducer of the epithelial-mesenchymal transition process during fibrotic remodeling of airways (21). Both, TGF-β1 and TGF-β2 have been shown to be increasingly expressed during airway hyperreactivity (AHR), especially in eosinophils (22) and macrophages (23); in case of TGF-β2, in epithelium (24) and neutrophils. The amount of TGF-β1 in bronchoalveolar lavage (BAL) is increased in AA and both TGF-β1 and -2 levels are increased upon segmental allergen challenge of the lung (25). TGF-β is part of the regulatory mechanisms of Tregs (26), which can keep the potentially pathogenic IL-4-producing Th2 cells under control and thus avoid AAI (27). The production of IL-4 by Th2 cells is also observed in healthy individuals (28). Therefore, IL-4 cannot be alone acting as a disease checkpoint. In contrast, IL-9-producing T helper cells (Th9) were recently identified as critical subset in AA (29). In fact, increased IL-9-secretion was observed in BALF and lung tissue of AA patients (30). Allergen-induced-IL-9 was described to directly induce mucus production (31), support *de novo* mast cell generation and their proliferation *in situ* (32). It further serves as chemoattractant for recruitment of inflammatory cells to sites of inflammation (33). For Th9 cells both TGF-β-dependent (34) and –independent (35, 36) mechanisms of induction were described. TGF-β can directly induce Th9 cells without the need of another T-cell subset by epigenetic mechanisms (37, 38). Importantly, TGF-β primes together with IL-4 the production of IL-9 in PPARγ^+^ Th2 cells (39), as also shown for the induction of Th17 and Treg cells (40).

Beyond systemic autoimmune inflammation, TGF-β signalling was found to be essential for pathological T cell-mediated effects in a variety of diseases, such as experimental autoimmune encephalomyelitis, a multiple sclerosis model (7). Further, in lymphopenic disease induced by early abrogation of TGF-β signalling in T cells a pronounced involvement of the lung was a consistent observation (6, 7). Nevertheless, while this was connected to the extreme situation of lymphopenia, it remained an open question to which extent the TGF-β pathway would be involved in the homeostasis of T cells in AAI. To address this question, we used our system for inducible ablation of TGF-βRII in Th cells and interrogated the role of TGF-β signaling in a mouse model of AAI. While we confirmed a role of TGF-β signaling in T cells not only for the disease course but also in the development or recruitment of inflammatory T cells in AAI, we found astonishingly little involvement in Treg homeostasis. To test whether similar observation could be made in human rhinitis patients we took advantage of induced sputum as a non-invasive window to lung pathology in allergic patients and in context of allergen-specific immunotherapy (AIT). We confirmed opposite regulation of TGF-β with respect to IL-4 and IL-9 by disease and treatment and found no influence of local TGF-β signaling on Treg cells. Taken together, in contrast to our expectation, TGF-β signaling had a direct influence on pathogenic T cell compartments. This appeared to be directly responsible for the establishment of AIT mediated tolerance. In contrast, Treg cells did not show dependence on TGF-β in airway inflammation in mouse and human.

## Results

### Reduced Airway Hyperreactivity and Lung Cell Infiltration in Allergic iCD4TGFBR2 Mice

**Figure 1:**
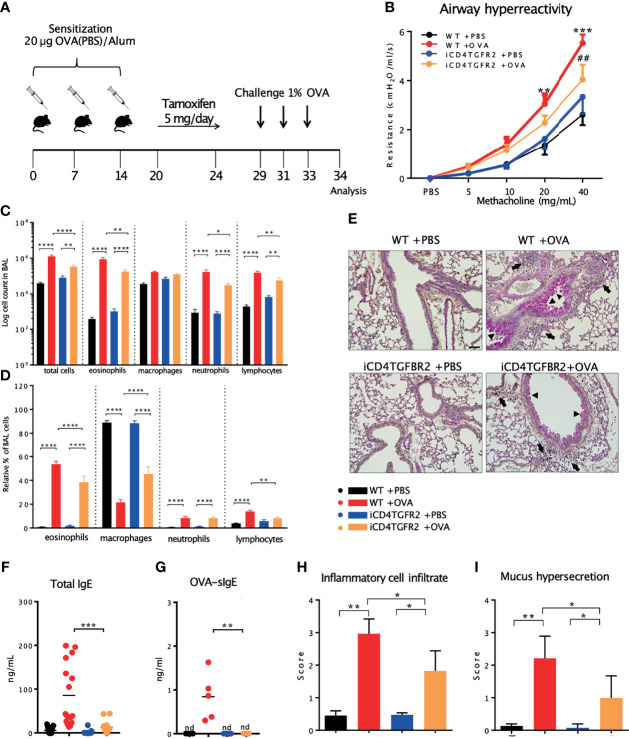
Impact of *Tgfbr2* ablation on lung inflammatory infiltrate and AHR. **(A)** Scheme of AAI induction, challenge and tamoxifen treatment. **(B)** Airway hyperreactivity measured in intubated, mechanically ventilated animals following methacholine provocation. n= 5 - 9/group; **p < 0.01; ***p < 0.001 vs WT+PBS; ^##^p < 0.01 vs WT+OVA at same methacholine concentrations (two-way analysis of variance (ANOVA) with Bonferroni’s *post-hoc* test). **(C)** BAL total cell number and **(D)** relative population size of mice from the four experimental groups. **(E)** PAS-staining of lung sections from the four experimental groups. Arrows: inflammatory infiltrate; arrowheads: mucus hypersecretion; scale bar: 50 µm. **(F, G)** Levels of total and OVA-specific Immunoglobulin E (tIgE: n=11-16/group; OVA-sIgE: n=5/group) measured in plasma samples (two-tailed Mann-Whitney U test). **(H, I)** Histological scores of **(H)** Inflammatory cell infiltrates and **(I)** mucus production (mean ± SD; n=5/group). **(C, I)** Representative of three independent experiments each with 5 mice per group. *p < 0.05, **p < 0.01, ***p < 0.001, ****p < 0.0001, nd, not detected.

We used OVA-induced AAI in a mouse model to address the question whether TGF-β is the cytokine that tips the balance between inflammatory or regulatory T cells upon allergen exposure. Because of the requirement of TGF-β for development of these pro and anti-inflammatory cell types it was an open question whether abrogation of TGF-β signaling would lead to an ameliorated or worse AAI disease course. We restricted our analysis to the time period after the sensitization phase, to exclude the known effects of TGF-β signalling on T cell receptor signal strength during the priming phase of our experiment. In our setup we compared presence versus absence of TGF-β signaling by use of tamoxifen-facilitated Cre-loxP-mediated deletion of *TGFBR2* in CD4^+^ cells (**Figure 1A**). These iCD4TGFBR2 mice (7) were compared to wildtype (WT) C57BL/6/J animals. Twenty-four hours after the last allergen challenge, we observed decreased AHR to methacholine in absence of TGF-β signaling (**Figure 1B**), showing a role of this pathway in T cells during the effector phase of AAI. The reduction of disease upon *Tgfbr2* ablation went along with significantly decreased number of leukocytes, specifically eosinophils, neutrophils, and lymphocytes but not macrophages in BAL, with the most prominent change in the eosinophil population (**Figures 1C, D**). PAS-staining of lungs revealed decreased perivascular and peribronchiolar inflammatory cell infiltration (**Figures 1E, H**) and lower mucus hypersecretion following AAI upon *Tgfbr2* ablation (**Figures 1E, I**), confirming the ameliorated clinical outcome. These local changes to the severity of AAI by abrogation of TGF-β signaling in CD4^+^ T cells were reflected in serum, in which the levels of total IgE (**Figure 1F**) and OVA-specific IgE (**Figure 1G**) were drastically reduced. Without induction of AAI, induced Th cell specific *Tgfbr2* ablation did not result in any of the above-described outcomes, hence highlighting the critical role of TGF-β signaling during the challenge phase of established AAI.

### Cytokine and T Cell Response Upon Ablation of *Tgfbr2* in CD4^+^T Cells During AAI

**Figure 2:**
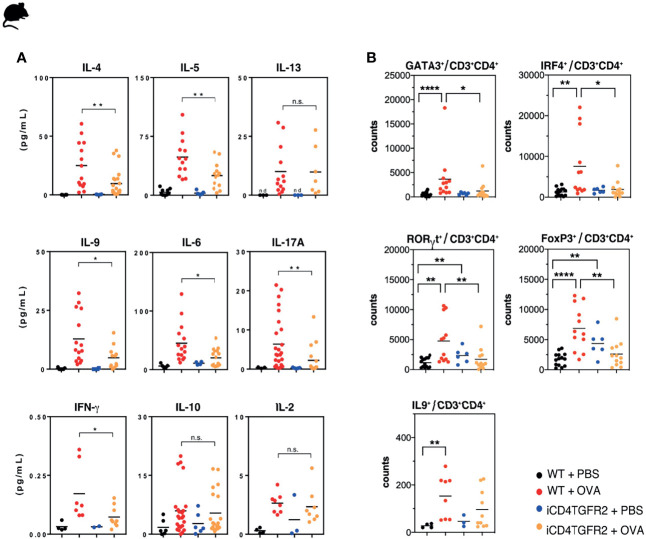
Impact of *Tgfbr2* ablation on lung cytokine production and T cell subsets. **(A)** BAL fluid of the indicated experimental groups was analyzed using electrochemiluminescence assay. Each data point represents an individual mouse. Data is compiled from one or two independent experiments (n=5-15/group). *p<0.05, **p<0.01, n.s. not significant (t-test). **(B)** Flow cytometric analysis of lungs focusing on distinct T cell subsets, performed 24h after the last OVA-challenge. Total Th2 (GATA3^+^), Th9 (IRF4^+^; IL-9^+^), Th17 (RORγt^+^) and Treg (FoxP3^+^) cells within the CD3^+^CD4^+^T cell population in non-allergic WT+PBS and iCD4TGFBR2+PBS mice and allergic WT+OVA and iCD4TGFBR2+OVA mice were assessed. Each data point represents an individual mouse. Data are representative of two independent experiments (n=6-13/group). *p < 0.05, **p < 0.01, ****p < 0.0001; n.s, not significant (two-tailed Mann-Whitney U test).

To better understand how T cell-specific abrogation of TGF-β signaling before the challenge phase was ameliorating AAI, we determined cytokine levels in BAL fluid. As expected, AAI resulted in a local increase of all measured cytokines in WT animals (**Figure 2A** and **Table S1**). In absence of *Tgfbr2* signals within Th cells we observed significantly less IL-4, -5, -6, -9, -17A, IFN-γ (**Figure 2A** and **Table S1**), and TNF-α (**Figure S1A**), while IL-1β, CXCL-1, CXCL-2, CCL-2, and CCL-3 showed the same trend but did not reach statistical significance (**Figure S1A**). Similarly, serum levels of IL-9 appear lower following *Tgfbr2* ablation in allergic animals (**Figure S1C**), but this difference did not reach significance. IL-2, IL-10, and IL-13 levels in BAL remained unaffected by *Tgfbr2* ablation (**Figure 2A**).

Analysis of the specific cytokine response by splenocytes after *in vitro* restimulation with OVA revealed significantly reduced production of IL-2 and IL-5 after *Tgfbr2* ablation. IL-4, IL-6, and IL-9 appear to show the same direction of response (**Figures S1D–E**). The specificity of these immunological effects is supported by the unchanged TNF-α secretion (**Figure S1E**).

We next assessed whether the changes in the cytokine levels found in BAL and serum were associated with the numbers of the respective T cell types located in the lung. We found decreased frequencies of GATA3^+^ Th2 cells and RORγt^+^Th17 following *Tgfbr2* ablation in allergic animals (**Figure 2B**). Also, the number of T cells expressing IRF4, a transcription factor active in various T cell lineages but absolutely required for Th9 development (41, for review see: 42), was reduced. However, the number of IL-9^+^ T cells was only slightly decreased. Both, AAI induction or *Tgfbr2* ablation alone led to an increased fraction of FOXP3^+^ Tregs in the lung (**Figure 2B**), as would be expected from a previous report (7, 43, 44 **)**. Unexpectedly, we did not find an additive or synergistic effect of combined AAI induction and *Tgfbr2* ablation (**Figure 2B**) with respect to Treg cell numbers in the lung.

We also found the fraction of lung CD8^+^ T cells to be increased in absence of TGF-β signaling in CD4^+^ T cells, while CD4^+^ T cells themselves were unchanged, as also the γδ T-cell percentages (**Figure S1F**).

Taken together, abrogated TGF-β signaling resulted in reduced responses of the Th2, Th9, and Th17 lineages during AAI while Treg cells expanded independently of AAI.

### Gene Network and Transcriptional Analysis

**Figure 3:**
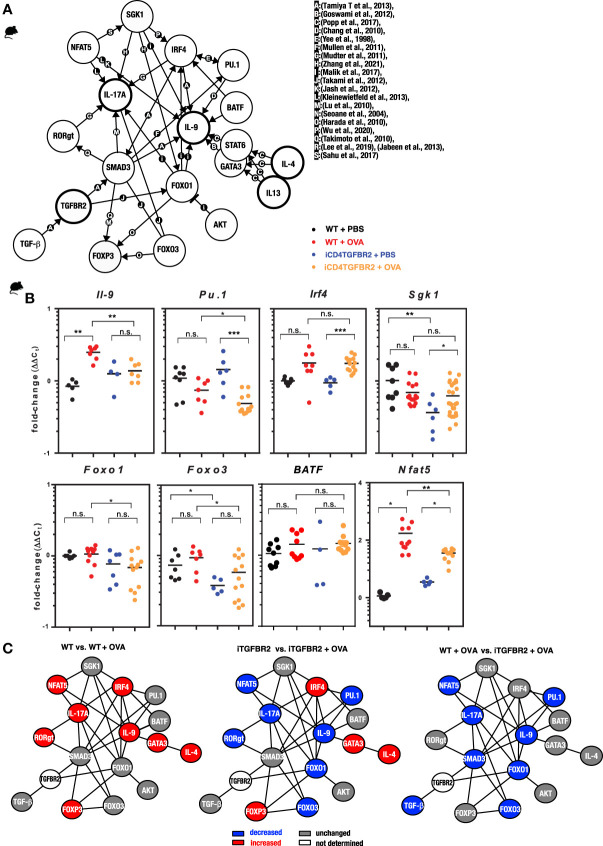
Impact of *Tgfbr2* ablation on Th-subset transcription factors and signal molecules. **(A)** Illustration of the complex interaction of genes in the regulation of T cell differentiation in allergy. The network indicates known molecular interactions or dependencies (either direct binding or gene regulation) and was inspired by the ImmunoNet (immunet.princeton.edu) (45) and extended manually by systematic literature research. The current knowledge was compiled in a gene regulation network of T_H_2, T_H_9 and T_H_17 networks. The references for the network were manually added and are as follows: [a: (46), b: (47), c: (48), d: (49), e: (50), f:(51), g: (52), h (53), i: (54), j: (55), k: (56), l: (57), m: (58), n: (59), o: (60), p: (61), q: (62), r: (63), (64), s: (65)]. **(B)** CD4^+^ T cells were isolated from digested lungs of non-allergic and allergic WT and iCD4TGFBR2 mice and analyzed for IL-9 expression as well as T_H_9 and T_H_2 relevant transcription factors and signal molecules using quantitative RT-PCR. Expression levels of *Il9, Pu.1, Irf4, Sgk1, Foxo1, Foxo3*, *Batf* and *Nfat5* were normalized to *Gapdh* house-keeping gene and relative changes were represented as 2^−ΔΔCT^ (ΔΔC_T_=ΔC_T_−ΔC_Control_). Data is compiled from two independent experiments (n= 5-13/group). *p < 0.05, **p < 0.01, ***p < 0.001, n.s. not significant (t-test). **(C)** Comparisons of gene regulation network of T cells. Blue indicates reduced gene expression, while red indicates increased and gray unchanged gene expression of the respective factor for the respective comparison.

To further understand T cell differentiation in allergy, a gene interaction network was assembled (**Figure 3A**). Key transcription factors of pro-inflammatory T cell subsets that directly or indirectly relate to TGF-β signaling are IRF-4 and PU.1, whereby IRF-4 is known to synergize with PU.1 (50). Th2, Th9 and Th17 are not only linked by IRF4 and SMAD3, but also FOXO1 and FOXO2 are regulating pro-inflammatory T cell subsets (55). *Pu.1* appears to be essential for *IL9* expression (49), and IRF4 can interact with NFATc2 to enhance *Il-4* gene expression (66) and with SMAD-3 to promote *Il9* expression (67). The IL-4-and TGF-β-inducible factors BLIMP-1 (not shown) inhibits IL-2 (68) and IL-9 (69). IRF4 also facilitates expression of the *IL-17* gene (52). To assess the different components of this network we performed expression analysis on CD4^+^ T cells from lungs of animals of our four experimental groups. Allergic inflammation enhanced the protein levels of IL-4, IL-9, IL-13 and IL-17 (**Figure 2A**) and mRNA levels of *Il9*, *Nfat5*, whereas *Foxo1* and *Foxo3*, *Spi1* (coding for PU.1), *Sgk1* and *Batf* remained unchanged (**Figure 3B**). Ablation of *Tgfbr2* decreased expression of *Il9*, *Pu.1*, *Foxo1*, *Foxo3* and *Nfat5* in diseased animals and of *Sgk1* and *Foxo3* in control animals. The current knowledge of gene regulation networks of pro-inflammatory Th2, Th9, and Th17 cells is overlapping or linked and some of these factors are connected to TGF-β signaling (**Figure 3C**). To obtain a schematic overview on gene regulation we assessed mRNA levels of these factors in the lungs with and without induction of AAI. Allergic inflammation in WT animals enhanced expression level of some Th2, Th9, Th17, and Treg and actors including IRF4, while the Th9 transcription factor PU.1 remained unaffected (**Figure 3C**, left). Allergic inflammation in *Tgfbr2*-ablated animals decreased the expression of Th17 and Th9 factors, while Th2 and Treg factors remained unchanged (**Figure 3C**, center and right) compared to WT (left). Interestingly, the IRF4 increase induced by allergic inflammation (left) is not affected by *Tgfbr2* ablation (center and right) despite the relationships of IRF4 to the TGF-β signaling.

Taken together, ablation of *Tgfbr2* seems to affect the transcriptional network of Th17 and Th9 cells, leaving the Th2 and Treg networks largely untouched.

### Effect of AIT on TGF-β and T Cell Lineage Specific Cytokines in Induced Sputum of Allergic Patients

In our AAI mouse model, we observed very specific effects of *Tgfbr2*-ablation on cytokine production and respective T cell lineages in the lung and proved the influence of TGF-β on local allergic airway reactions in established AAI. To confirm the relevance of these results for human allergy, we next investigated immune cells of the lower airways of rhinitis patient without and with asthma comorbidity in the context of AIT. Our cross-sectional cohort consisted of 26 healthy controls and 38 allergic rhinitis patients (AR), 19 of these with asthma comorbidity (AA; **Table S4**). Ten AR patients and nine AA patients received AIT, while nine untreated AR and ten untreated AA were assigned to the untreated groups.

**Figure 4:**
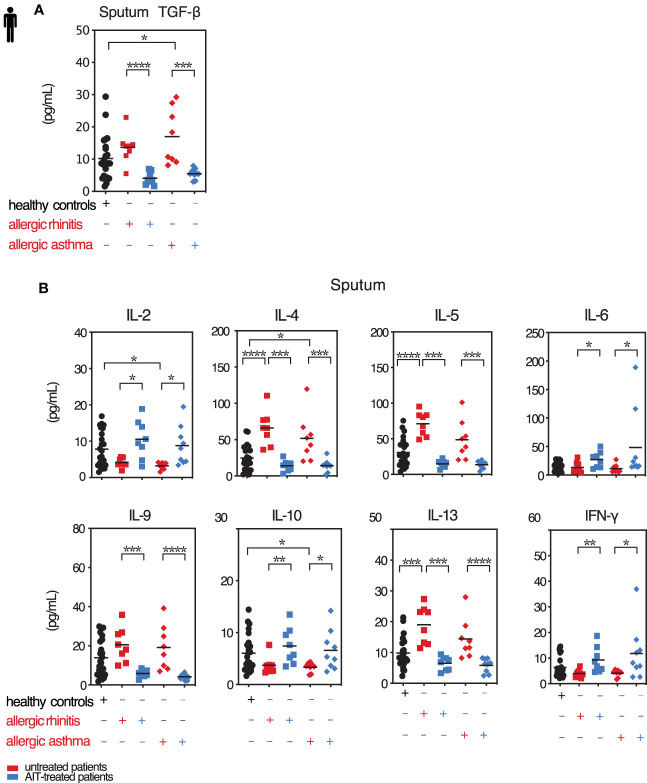
Local TGF-β is decreased along with pro-inflammatory cytokines upon AIT. Levels of selected cytokines in sputum of treated and untreated AR and AA patients and healthy controls. **(A)** Secreted TGF-β cytokine levels in induced sputum of allergic rhinitis and allergic asthma patients receiving (blue colored) or not (red colored) AIT as well as of control individuals were analyzed by LEGENDplex. Data presented by individual values and mean. **(B)** Levels of secreted cytokines IL-2, IL-4, IL-5, IL-6, IL-9, IL-10, IL-13 and IFN-γ detected in sputum supernatants of the groups as in **(A)** assessed by LEGENDplex. Data presented by individual values and mean. *p < 0.05, **p < 0.01, ***p < 0.001, ****p < 0.0001; statistical significance was determined by Kruskal-Wallis tests and only when medians across patient groups varied significantly, multiple single comparisons were performed using two-tailed Mann-Whitney U tests.

In induced sputum samples from our patient group, we observed that AIT reduced back to baseline the elevated levels of TGF-β found both for AR and AA patients *in season* of natural pollen exposure (May-July, **Figure 4A** and **Table S5**). We also observed that sputum levels of TGF-β1 showed a positive correlation with total IgE (r=0.3488, *p*=0.0189; data not shown). Hence, allergic patients under AIT reveal a situation like the one artificially obtained by *Tgfbr2* ablation in the mouse model. AIT reduced back to baseline the cytokines IL-4, IL-5, IL-9, and IL-13, thus the classical mediators of Th2 and Th9 cells respectively (**Figure 4B** and **Table S5**). The levels of IL-2, IL-6, IL-10, and IFN-γ, hence pan-T cell, inflammatory, Treg, and Th1 cytokines, respectively, showed the opposite reaction with higher sputum levels upon AIT (**Figure 4B**). While the effect sizes were smaller, these observations were confirmed *out of seaso*n (October-January, **Figure S2A**).

Taken together, following AIT in AR and AA patient airways TGF-β levels are reduced in parallel with those of Th2 and Th9 cytokines.

### Decreased Th2 and Th9 and Increased Treg Cells Upon AIT

**Figure 5:**
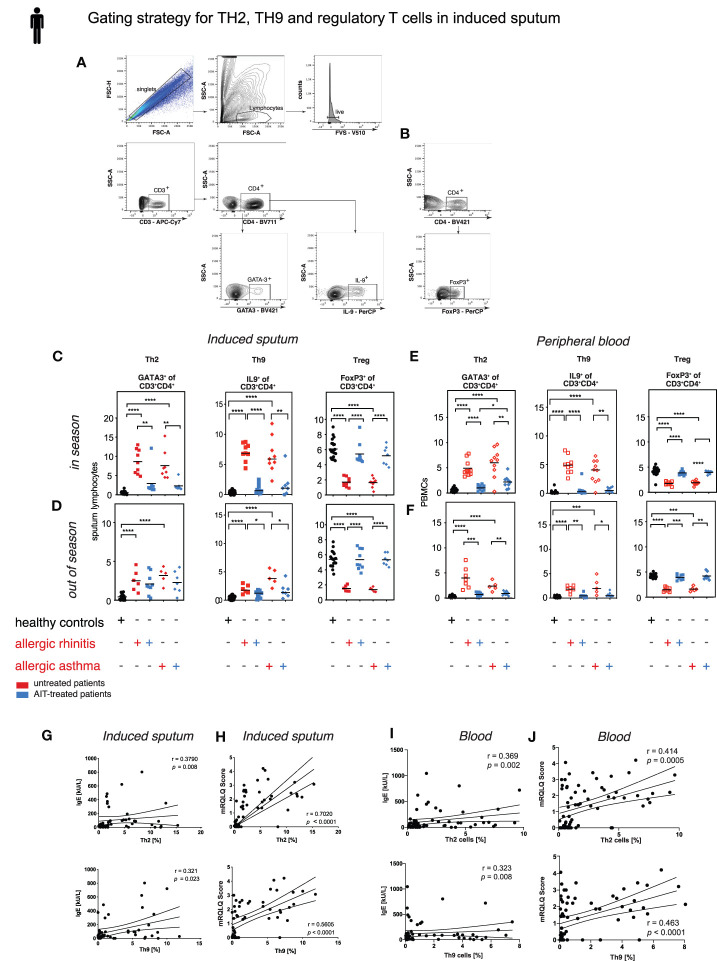
Treg cells increase and Th9 as well as Th2 cells decrease upon AIT. Th2, Th9, and Treg cell frequencies in sputum of treated and untreated AR and AA patients and healthy controls. **(A)** Gating strategy for Th2 and Th9 and **(B)** Treg cells in induced sputum. **(C, D)** Flow cytometric analysis of sputum derived T cell subpopulation Th2, Th9 and Tregs in healthy subjects, AR and AA patients as described in *in season* **(C)** and *out of season* **(D)**. **(E, F)** Flow cytometric analysis of peripheral blood derived T cell subpopulation Th2, Th9, and Treg cells in healthy subjects, AR and AA patients as described in *in season* **(E)** and *out of season* **(F)**. Data presented by single patient values and mean. Statistical significance was determined by Kruskal-Wallis tests and only when medians across patient groups varied significantly, multiple single comparisons were performed using two-tailed Mann-Whitney U tests. *p < 0.05, **p < 0.01, ***p < 0.001, ****p < 0.0001. **(G, H)** Correlation of sputum T_H_2 and T_H_9 cell frequencies with total serum IgE **(G)** and symptom score mRQLQ **(H)**. **(I, J)** Correlation of peripheral blood derived T_H_2 and T_H_9 cell frequencies with total serum IgE **(I)** and symptom score mRQLQ **(J)**. Two-sided Spearman test was used to calculate the correlations.

Since TGF-β has distinct effects dependent on specific tissue context, we quantified the frequency of Th2 cells (GATA3^+^ CD3^+^CD4^+^), Th9 cells (IL9^+^CD3^+^CD4^+^), and Treg cells (FoxP3^+^ CD3^+^CD4^+^) in induced sputum samples and PBMCs by flow cytometry (**Figure 5**). *In season*, frequencies of sputum Th2 and Th9 cells were strongly reduced in treated patients compared to untreated patients, both in sputum (**Figure 5C**) and in blood (**Figure 5E**). Percentages of Treg cells were, however, increased after AIT (**Figures 5C, E**). Hence, for all three T cell lineages frequencies were basically normalized by AIT. Similar effects, albeit less pronounced, could be observed *out of season* (October-January) of natural pollen exposure (**Figures 5D, F**). Surprisingly, *out of season* the amelioration of Th2 frequencies by AIT in sputum was very small in contrast to the Th9 frequency change (**Figure 5D**), while in blood the effect size remained large (**Figure 5F**). The size of the Th2 and Th9 subsets in sputum and blood presented only weak or no correlation to serum IgE levels (**Figures 5G, I**), whereas the symptom score mRQLQ, most relevant as therapy outcome parameter, was strongly positively correlated to the size of both Th subsets (**Figures 5H, J**). These results demonstrate that AIT modulates the local and systemic abundance of pro-allergic Th2 and Th9 cell subtypes, while Treg cells and local TGF-β were increased.

## Discussion

The exact role of the TGF-β pathway during allergic airway disease awaits further delineation. With the available data TGF-β can be postulated to play contradictory roles, pro- and anti-inflammatory. This unclarity about TGF-β ´s role in AAI may be one reason why the pathway has so far been excluded from consideration as a drug target. Therefore, our study was aimed at dissecting these contradictory pro- and anti-inflammatory roles of TGF-β with a focus on inflammatory and regulatory T cells at the site of allergic airway inflammation. We started our investigation by using a murine model of AAI with induced *Tgfbr2* ablation in CD4^+^ T cells (7). We demonstrate that mice with *Tgfbr2* ablation induced between sensitization and challenge phase presented reduced clinical features of AAI, confirmed by strongly ameliorated histological and functional disease parameters. Hereby, the study shows that during AAI TGF-β-signaling in CD4^+^ T cells is an important factor for allergogenic Th2, Th9 and Th17 cell activity *in vivo*, while the Treg compartment remained largely unaffected. Th2, Th9 and Th17 cell recruitment upon allergen challenge (70) was reduced in absence of TGFBR2 along with respective transcription factors PU.1 (Th9), GATA3 (Th2), and NFAT5. NFAT5 is an osmosensitive transcription factor that negatively regulates IL-17 and IFN-γ gene expression (71) and enhances Th2 response (72). Moreover, the transcription factor FOXO1, which was recently described to be essential for Th9 cell differentiation and IL-9 production, was clearly reduced upon *Tgfbr2* ablation, correlating with reduced airway allergy (54, 73, 74). This specific impact of *Tgfbr2* depletion on T cell subsets during AAI was not limited to the respiratory system, but also extended to a systemic response measured in splenocytes. In line with these reduced sizes of pathologically relevant T cell lineages, disease activity was also ameliorated in terms of infiltrating myeloid cells and production of total and specific IgE. Treg cells were shown to be able to suppress AAI in various models (75, 76). Yet, in our model of induced ablation of TGF-β signaling on T cells we did not observe an effect on the size of the local and systemic Treg compartments during AAI. Hence, it appears likely that the Treg compartment size is not responsible for the reduced disease severity and associated cellular and molecular changes in *Tgfbr2*-ablated mice. Another example of such Treg independent suppression of AAI was reported for treatment with the TLR2 agonist Pam3CSK4, although it involved different mechanisms as seen by us (77).

Taken together, absence of TGF-β signaling by CD4^+^ T cells after sensitization did not interrupt the immune control by the Treg cell compartment, while we clearly confirmed a major role for the Th9-, Th2-, and Th17-driven allergic response upon challenge. A limitation of our study is that that we could not restrict abrogation of TGF-β signaling to all the relevant allergogenic T cell lineages or Treg cells. Also, as we found first indications of reduced CD103 expression, an integrin regulating tissue-residency (10–13), this may reduce migration of T cells to the inflamed lung and their retention (78). Future studies are needed to explore the role of integrins in TGF-β dependent T cell populations and bone marrow chimaeras could dissect direct versus indirect effects of *TGFBR2* ablation on the differentiation of inflammatory T cell populations.

Similarly, to the murine AAI model, in human patients suffering from AR, treatment with AIT shows decreased levels of sputum TGF-β along with the pro-inflammatory type-2 cytokines. We took advantage of this treatment-induced TGF-β-modulation and monitored Treg, Th2, and Th9 cells. In the AIT treated patients we demonstrate that the seasonally induced levels of Th2/Th9 cytokines were strongly decreased, locally and systemically, while Th1, general inflammatory cytokines, as well as the suppressive cytokine IL-10, were increased. These observations were mirrored by the respective population sizes, again locally and systemically. While local sputum cytokine levels were not yet investigated in context of AIT, it was previously shown that AIT controls IL-9 expression in the upper airways (79), as well as Th2 frequency in biopsies (79) and peripheral blood (28). Interestingly, Th2 cells were also detectable in sputum of health individuals both *in-* and *off-season*. This finding confirms our previous studies, in which we reported IL-4 mRNA and secreted IL-4 protein at low, but detectable levels in the airways of healthy individuals (28).

While the presence of Treg cells was previously detected in sputum (80) we were surprised that Treg cells constituted the most abundant T cell subpopulation in the sputum of healthy individuals *in* and *off season*. Furthermore, untreated AR and AA patients consistently displayed strongly reduced Tregs frequency compared to healthy individuals even *off-season*. In contrast, the AIT treatment restored Treg cells percentages, locally and systemically, even though TGF-β levels were reduced by AIT even below levels of healthy individuals. This result indicates that AIT induces a special condition of immunosuppression and is not restoring natural allergen tolerance in the first years of treatment. Overall, it appears that TGF-β plays a major role in the allergic response but not its suppression. Although the study was not powered to quantify treatment success, the symptom load was reduced by AIT treatment and correlated strongly with the local, and somewhat less with the reduced systemic percentages of Th2 and Th9 cells.

A reduction of Tr1 cells in the context of allergic airway disease has been reported previously and may be represented in our dataset by the reduction in IL-10 in induced sputum of allergic patients (81).

Taken together, our data demonstrate that TGF-β is a major player in allergic airway inflammation by reprogramming T cells into effector T cells. Since Treg cells remain unaffected (mouse) or are even increased (patients with AIT) by reduced levels of TGF-β signaling, targeting of the TGF-β pathway might therefore not only suppress tissue-remodeling processes but also target pro-inflammatory T cells, without the risk of affecting immune tolerance adversely.

## Methods

### Animals and Murine Model of AAI

C57Bl/6J mice, originally obtained from Charles River, and CD4Cre^ERT2^ *Tgfbr2* ^fl/fl^ mice (iCD4TGFBR2) were bred at the animal facility at the Institute of Comparative Medicine of the Helmholtz Centre Munich. Generation and characterization of iCD4TGFBR2 mice was described elsewhere (7). All mice were co-housed under specific pathogen-free conditions in individually ventilated cages (VentiRack) and fed by standard pellet diet (Altromin Spezialfutter GmbH & Co. KG) and water *ad libitum*. Both female and male mice aged 6-8 weeks were used for the experiments. To induce AAI an established ovalbumin-sensitization model was used (82). Briefly, iCD4TGFBR2 and WT C57Bl/6J mice were sensitized by i.p.-injections of 20 µg ovalbumin (OVA; grade V; Sigma-Aldrich) in phosphate buffered saline (PBS) adsorbed to aluminum hydroxide (2.5 mg, ImjectAlum) on days 0, 7 and 14. Non-allergic control mice received the same volume of PBS in alum. The assignment to the two different groups occurred randomly. On days 29, 31 and 33 all mice were aerosol-challenged for 20 minutes with 1% OVA in PBS delivered by a Pari-Boy nebulizer (Pari), (82). The experimental protocol is depicted in **Figure 1A**. Blood samples were taken before sensitization and at the end of the experiment. Animals were sacrificed twenty-four hours after the last OVA challenge. The study was conducted under federal law and guidelines for the use and care of laboratory animals and was approved by the Government of the District of Upper Bavaria and the Animal Care and Use Committee of the Helmholtz Center Munich (approval number: 55.2-1-54-2532-75-2012).

### Tamoxifen Treatment

To ablate the subunit 2 of the heterodimeric TGF-β receptor, mice were treated with tamoxifen (TM, tamoxifen-free base, Sigma-Aldrich) after the sensitization phase. Tamoxifen was suspended in 100% ethanol to 1 g/ml, vortexed, and mixed with corn oil (Sigma-Aldrich) to a final concentration of 100 mg/ml. Before *in vivo* administration, the solution was heated to 37°C until it was properly dissolved. On day 20-24, 50 µl (5 mg) tamoxifen per day were administered by intra-gastric gavage in both iCD4TGFBR2 and WT control mice (7).

### Measurement of Airway Hyperreactivity

AHR to methacholine (Mch; Sigma-Aldrich) was measured 24 h after the last OVA challenge in intubated, mechanically ventilated animals (n = 6–10/group; Data Sciences International (DSI), as previously described (83). Briefly, animals were anesthetized by an intraperitoneal injection of Ketamine (100 mg/kg) and Xylazine (5 mg/kg) in PBS. After cannulation of the trachea and starting mechanical ventilation, the animals were challenged with increasing methacholine (Mch) concentrations, using an in-line nebulizer (5 μl Mch solution in PBS delivered for 30 seconds at the following concentrations: 0, 5, 10, 20 and 40 mg/ml). Data were recorded using the FinePoint software v2.4.6 (DSI). The highest values of respiratory system resistance (R) were recorded every 5 seconds during the data recording interval set at 3 min after each Mch level. The heart rate of each animal was continuously monitored using an ECG device connected with three subcutaneous electrodes throughout the entire experiment (DSI).

### Analysis of Bronchoalveolar Lavage, Lung Histology and Serology

BAL and evaluation of inflammatory cell infiltration were performed as described previously (82). Aliquots of cell-free BAL fluid were used to measure cytokines and chemokines *via* mesoscale technique using two different kits (V-Plex proinflammatory panel 1 mouse kit and V-Plex cytokine panel 1 mouse kit; MesoScaleDiscovery) according to manufacturer’s instructions. Total and ovalbumin-specific IgE were measured in serum samples by ELISA as described previously (84). IL-9 was measured in serum samples using mouse-IL-9 ELISA (BioLegend). For lung histology, after BAL, the lungs were excised and the left lobe fixed in 4% buffered formalin and embedded in paraffin. Sections of 4µm thickness were stained with hematoxylin-eosin (H&E) and periodic acid Schiff (PAS). Mucus hypersecretion and inflammatory cell infiltration were graded in a blinded fashion on a scale from 0 to 4 (0=none, 1=mild, 2=moderate, 3=marked, 4=severe), reflecting the degree of the pathological alteration (82).

### Isolation and Analysis of Leukocytes From Lung Tissue

Lungs were excised, cut into small sized pieces and digested in RPMI medium supplemented with 100 µg/ml DNAse (Sigma-Aldrich) and 1 mg/ml Collagenase Type 1A (Sigma-Aldrich) at 37°C. Digested lungs were filtered through a 70 µm cell strainer, pelleted (400 G, 4°C, 5 min) and resuspended in 6 ml 40% percoll in RPMI (v/v) solution, which was underlayed with 4 ml 80% percoll solution (GE Healthcare-Life Sciences). Tubes were centrifuged (1600 G, RT, 15 min) with brake set to 0. Lymphocytes were collected from the interphase and analyzed by flow cytometry.

### Isolation and Restimulation of Splenocytes

Spleens were excised and single cell suspensions were obtained and re-stimulated as previously described (85). Cells were washed and re-suspended in complete medium [RPMI 1640 supplemented with 10% FCS, 1% glutamine, 1% penicillin-streptomycin, 1% Na-pyruvate, 1% non-essential amino acids (Gibco, Life Technologies GmbH) and 50µM 2-β-mercaptoethanol (Sigma-Aldrich)], plated in 96-well at a concentration of 2x10^5^ cells/well and cultured for 72 h with medium alone or with OVA V (5 µg/ml; Sigma-Aldrich). Their supernatants were analyzed for cytokine expression using the V-Plex proinflammatory panel 1 mouse and V-plex IL-9 kit (MesoScaleDiscovery) according to manufacturer’s instructions.

### Patients

Specimen were taken from 26 healthy controls and 38 AR patients, of whom 19 received AIT (**Table S4**). All subjects completed a Rhinoconjunctivitis Quality of Life mini Questionnaire (mRQLQ), a lung function test and sputum induction. GINA scores were assessed from asthmatic subjects. Each participant provided written informed consent. The study was approved by the local ethics committee (5534/12). Among AIT-treated patients, 9 patients were additionally affected by asthma, 10 suffered of AR only. Asthmatic patients are represented as a subgroup of the rhinitis patients, if not otherwise indicated. Patients were considered as asthmatic based on previous physician’s diagnosis and with a reported history of shortness of breath, cough, chest tightness during natural allergen exposure and/or earlier documented positive bronchodilation test. All patients were in good health (FEV1% >70%) with a history of clinically significant hay fever during the grass-pollen season since more than two years. Patients of the AIT-treated groups received at least one year of AIT treatment. Thereby, grass-pollen allergic patients with a history of moderate-severe and chronic persistent allergic rhinitis as defined by ARIA (Allergic Rhinitis and its Impact on Asthma) criteria since >2 years during the grass-pollen season, a positive skin prick test wheal >3mm in diameter and grass pollen specific IgE-level above 0.70kU/L underwent subcutaneous grass-pollen AIT.

In addition, total IgE was measured from sera of all individuals included in this study. Peripheral blood samples from all subjects included in this study were drawn at the same time points as sputum samples and were analyzed by flow cytometry. For flow cytometric analysis, CPT tubes (BD biosciences) were centrifuged according to manufacturer’s instructions. Further, each sample was adjusted to 2.0x10^6^ cells and used for subsequent FACS staining. All procedures were performed in the Allergy Section, Department of Otolaryngology, TUM School of Medicine, Munich, Germany.

### Sputum Collection, Processing and Characterization

Collection and processing of sputum as well as differential cell counts was performed as previously described (86). Briefly, human participants first inhaled salbutamol and consecutively nebulized hypertonic saline at increasing concentrations of 3%, 4%, and 5% NaCl every 7 min. During this procedure, participants cleaned their noses and rinsed their mouth to reduce squamous epithelium cells in the samples. Sputum was processed within one hour of collection. The selected sputum plugs, which contained as little saliva as possible, were placed in a weighed Eppendorf tube and processed with 4x weight/volume of sputolysin working solution (Merck). Afterwards, 2x weight/volume of PBS was added. Samples were filtered through a 70 µm mesh and centrifuged for 10 min at 790 x g without break to remove the cells. Supernatants were stored at –80°C until further analysis. In addition, sputum cell slides were prepared for differential cell counts. Sputum samples were successfully collected from healthy controls (n=24; 92.3%) and allergic patients (n=34; 89.4%) once in and out of grass pollen season as previously described (86) and analyzed for secreted protein levels of TGF-β, IL-9, type-2 cytokines IL-4, IL-5, and IL-13, IL-10, type-1 cytokines IL-2, IFN-γ, and IL-6. Cytokine levels were analyzed by LEGENDplex TGF-β1 and multiplex human inflammation panel assay (BioLegend). All nine parameters were detectable in every sputum sample derived from patients with AR and AA and compared to healthy control subjects.

### Lung Function Testing in Humans

Baseline lung function was evaluated using a calibrated handheld pulmonary function testing device (Jaeger SpiroPro). The following parameters were recorded: vital capacity (VC), forced expiratory volume (FEV1), FEV1/VC, and maximum expiratory flow 25% (MEF 25%). Bronchodilator reversibility was tested after 400 µg of salbutamol.

### Total Serum IgE Measurement

Total serum IgE was assessed using diagnostic ImmunoCAP assays on a Phadia 100 device.

### Flow Cytometric Analysis of Human and Murine Samples

Human sputum cell or PBMCs samples were labeled without stimulation for flow cytometry with specific antibodies using the Foxp3/Transcription Factor Staining Buffer Set (eBioscience) according to manufacturer’s instructions.

For flow cytometric analyses of the murine samples, staining for transcription factors was performed by using Foxp3 Staining Buffer Set (eBiosciences), while cytokine staining was performed by using the fixation/permeabilisation solution Kit (BD Bioscience Cytofix/Cytoperm™) according to the manufacturer’s protocols. For intracellular cytokine staining, cells were stimulated for 4h with 50 ng/ml PMA (Applichem), 500 ng/ml Ionomycin (Thermo Fisher Scientific) and 1:1000 GolgiPlug (BD Bioscience). Flow cytometric analysis was performed using a BD LSRII Fortessa flow cytometer (BD Bioscience). Flow cytometric data of both human and mouse samples were analyzed with FlowJo software (FlowJo). For gating strategy of human sputum cells (0.5x10^6^ cells) or PBMCs (2.0x10^6^ cells) samples see **Figures 5A**, **S2B**; the murine gating strategy is shown in **Figure S1**. Antibodies used for flow cytometry are listed in **Table S2** (murine) and **Table S6** (human).

### Real-Time Polymerase Chain Reaction

Total RNA was extracted either from mouse total lung tissue after homogenization using the RNeasy Mini Kit (Qiagen GmbH) or from enriched mouse lung CD4^+^ T cells (CD4 T cell isolation kit, Miltenyi Biotec, Auburn, CA, USA), using RNeasy Micro Kit (Qiagen) according to supplier’s instructions. RNA was reverse-transcribed directly (RevertAid H Minus First Strand cDNA Synthesis Kit, Thermo Scientific) and quantitative real-time PCR was performed using SYBR Green PCR Kit Master Mix (Qiagen) and the LightCycler^®^480 System (Roche) as previously described (83). The used primer sequences are listed in **Table S3**. Each reaction was performed in duplicate in 284-well plates (Applied Biosystems) and turned into mean. The expression levels were normalized to GAPDH house-keeping gene and relative changes were represented as 2^−ΔΔCT^ (ΔΔC_T_=ΔC_T_−ΔC_Control_).

### Data Acquisition and Statistical Analysis

All experimental procedures and analyses were conducted by blinded research staff. For the mouse experiments, differences between the groups in AHR were evaluated with two-way analysis of variance (ANOVA) with Bonferroni’s *post-hoc* test. Otherwise, differences between two data sets were evaluated using unpaired two-tailed Mann-Whitney test or t-test. Data were expressed as mean ± S.D., if not otherwise indicated. All statistically significant differences were depicted as *p<0.05, **p<0.01, ***p<0.001, ****p<0.0001. Data were analyzed using Prism software version 6 (GraphPad software Inc.). The network map indicates known molecular interactions or dependencies (either direct binding or gene regulation) and was inspired by the ImmunoNet (immunet.princeton.edu) (45) and extended manually by systematic literature research. For the analysis of human samples, non-parametric statistical test were chosen, as the data points were not normally distributed. For **Figures 4**, **5**, Kruskal-Wallis tests were performed initially to avoid multiple testing, and, only when medians across patient groups varied significantly, multiple single comparisons were performed using two-tailed Mann-Whitney U tests. Two-sided Spearman correlation was used to correlate IgE or RQLQ with immune cell frequencies in **Figure 5**.

## Data Availability Statement

The original contributions presented in the study are included in the article/**Supplementary Material**. Further inquiries can be directed to the corresponding author.

## Ethics Statement

The studies involving human participants were reviewed and approved by Ethics committee of the Klinikum rechts der Isar (5534/12). The patients/participants provided their written informed consent to participate in this study. The animal study was reviewed and approved by Government of the District of Upper Bavaria and the Animal Care and Use Committee of the Helmholtz Center Munich (approval number: 55.2-1-54-2532-75-2012).

## Author Contributions

SM, FA, SH, TB, CS-W, and UZ developed the study and experimental layout. SM, FA, ES, BS, and JG realized experimental disease models and measurements of murine parameters. FA, SH, and TB supported the study on ethical permissions and funding. AC organized study part including human ethical approval and together with JK and MP the management of patient visits, patient information and sampling. Human samples were analyzed by IG, JU, JK, MP, FG, and UZ. The manuscript was written by SM, FA, TB, and CS-W as well as UZ. All authors contributed to the article and approved the submitted version.

## Funding

This study was supported by the German Center for Lung Research (DZL) to CS-W and UZ, Helmholtz Inflammation&Immunology (I&I) to CS-W, Grant of the German Research Foundation (DFG) No. 398577603 to CS-W and UZ, and No. TR22 to TB.

## Conflict of Interest

UZ received payment for manuscripts from Deutsches Aerzteblatt and funds for travel from the European Academy of Allergy and Clinical Immunology (EAACI) and Collegium Internationale Allergologicum (CIA). CS-W received support for research projects from PLS Design, LETI, Zeller AG, and Allergopharma and accepted honoraria for consultancy and seminars from LETI and Allergopharma. He also received travel support from EAACI. AC has consultant arrangements through Technical University Munich with Allergopharma, ALK-Abello, HAL Allergy, Mundipharma, and Lofarma; has conducted clinical studies and received research grants through Technical University Munich from Allergopharma, Novartis, the German Federal Environmental Agency, Bencard/Allergen Therapeutics, ASIT Biotech, and Zeller AG; has received payment for lectures from Allergopharma, ALK-Abello, and GlaxoSmithKline; has received payment for manuscript preparation from Bayerisches Ärzteblatt; and has received travel support from the European Academy of Allergy and Clinical Immunology (EAACI), DGAKI, and SMI.

The remaining authors declare that the research was conducted in the absence of any commercial or financial relationships that could be construed as a potential conflict of interest.

## Publisher’s Note

All claims expressed in this article are solely those of the authors and do not necessarily represent those of their affiliated organizations, or those of the publisher, the editors and the reviewers. Any product that may be evaluated in this article, or claim that may be made by its manufacturer, is not guaranteed or endorsed by the publisher.

## Abbreviations

AA, allergic asthma; AAI, allergic airway inflammation; AHR, airway hyperreactivity; AIT, allergen-specific immunotherapy; AR, allergic rhinitis; BAL, Bronchoalveolar lavage; BALF, Bronchoalveolar lavage fluid; ICI, Inflammatory cell infiltrate; i.p., intraperitoneal; Mch, metacholin; n.d., not detectable; OVA, ovalbumin; PBMC, peripheral blood mononuclear cell; PBS, Phosphate-buffered saline; mRQLQ, Rhinoconjunctivitis Quality of Life mini Questionnaire; tIgE, total Immunoglobulin E; Tregs, regulatory T cells; Th, T helper cells.
