# Supplementary figures and images for: TGF-β1 Drives Inflammatory Th Cell But Not Treg Cell Compartment Upon Allergen Exposure
Source: Front Immunol. 2022 Jan 7;12:763243. doi: 10.3389/fimmu.2021.763243 (PMC8777012; doi:10.3389/fimmu.2021.763243)

FigureS1

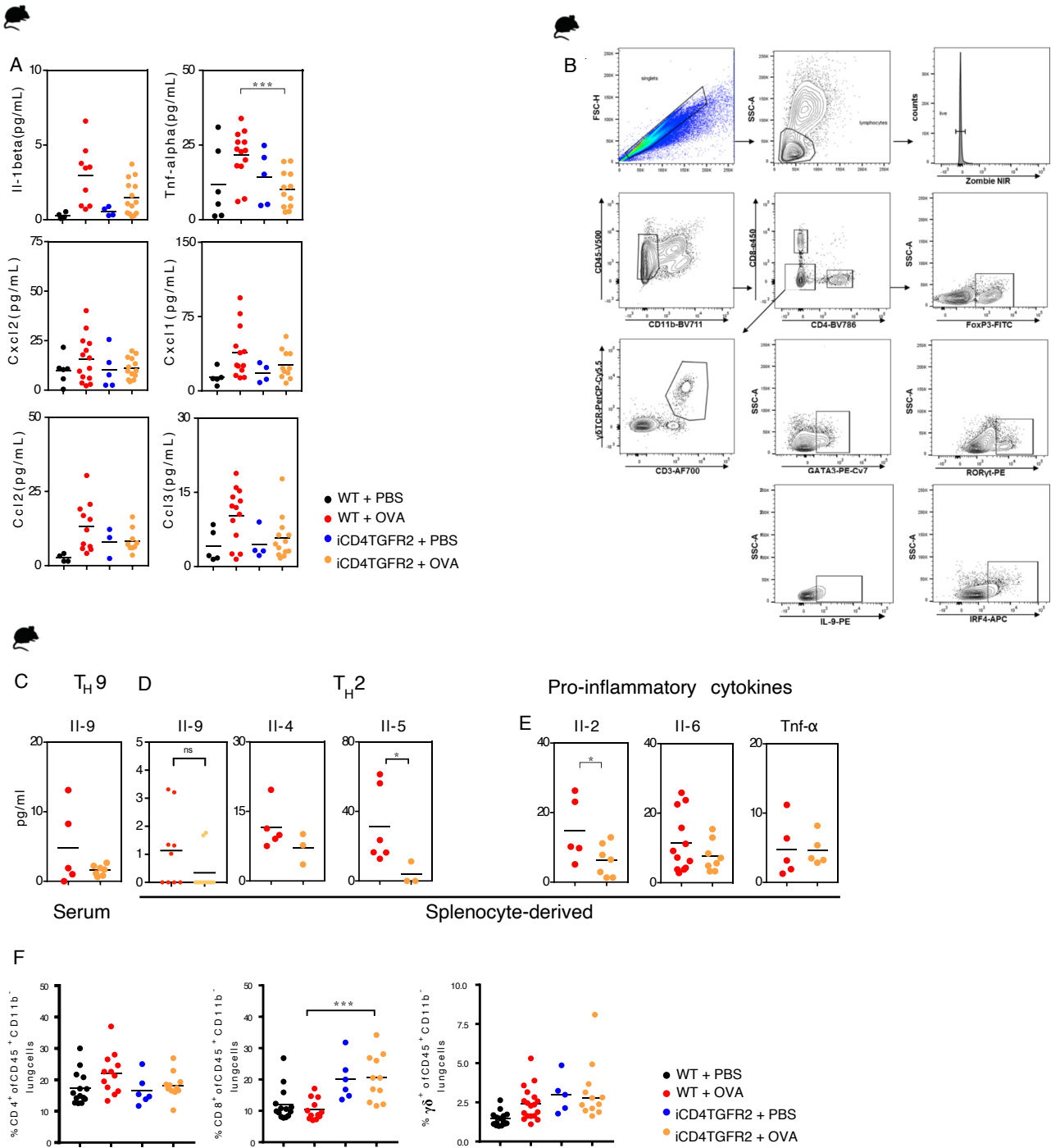

Supplement: Supplementary file 1 [file DataSheet_1.pdf]
