# Supplementary material for: TGF-β1 Drives Inflammatory Th Cell But Not Treg Cell Compartment Upon Allergen Exposure
Source: Front Immunol. 2022 Jan 7;12:763243. doi: 10.3389/fimmu.2021.763243 (PMC8777012; doi:10.3389/fimmu.2021.763243)

Figure S2

Sputum cytokine levels out of grass pollen season (October–January)

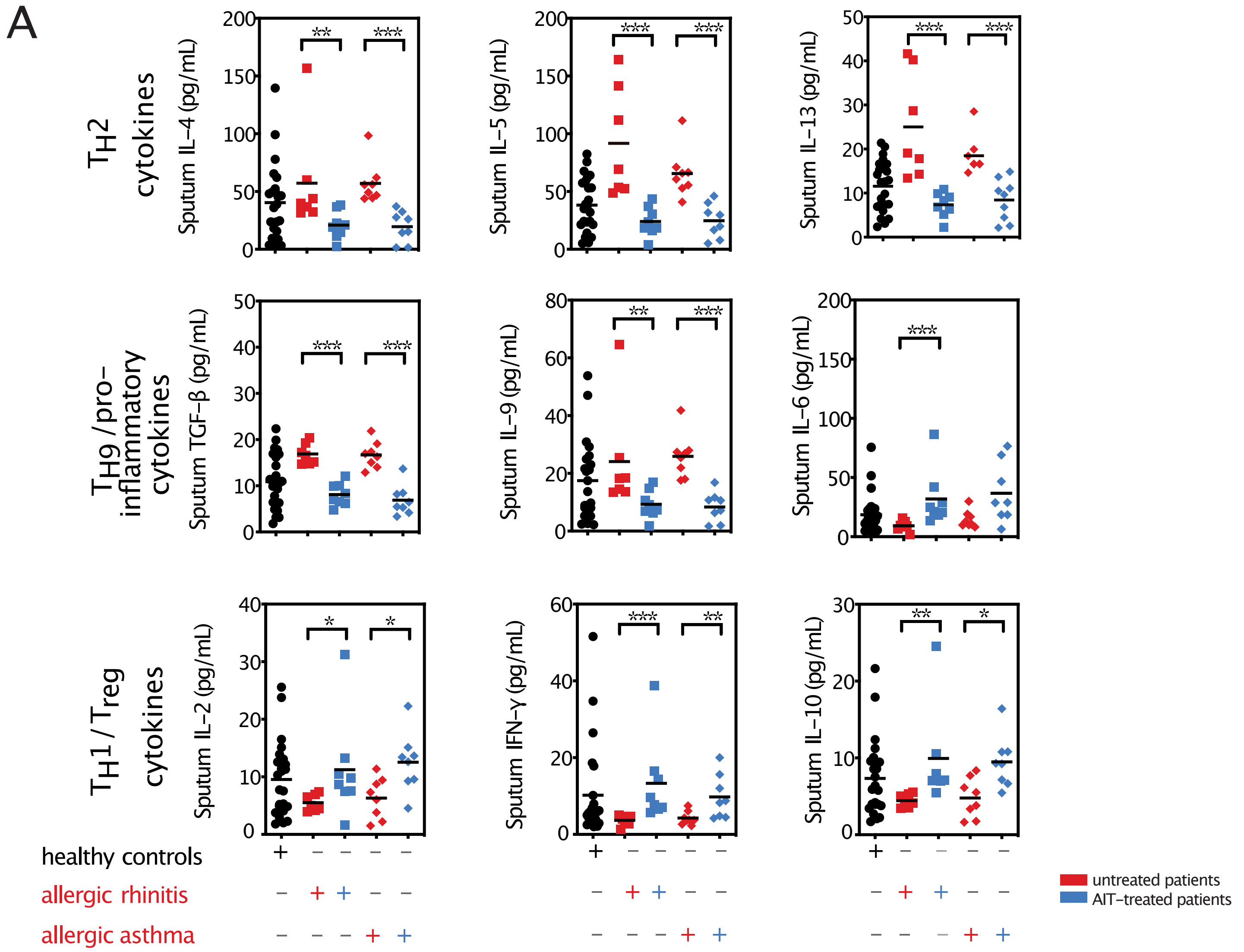

B Gating strategy for T_H2, T_H9 and regulatory T cells in peripheral blood

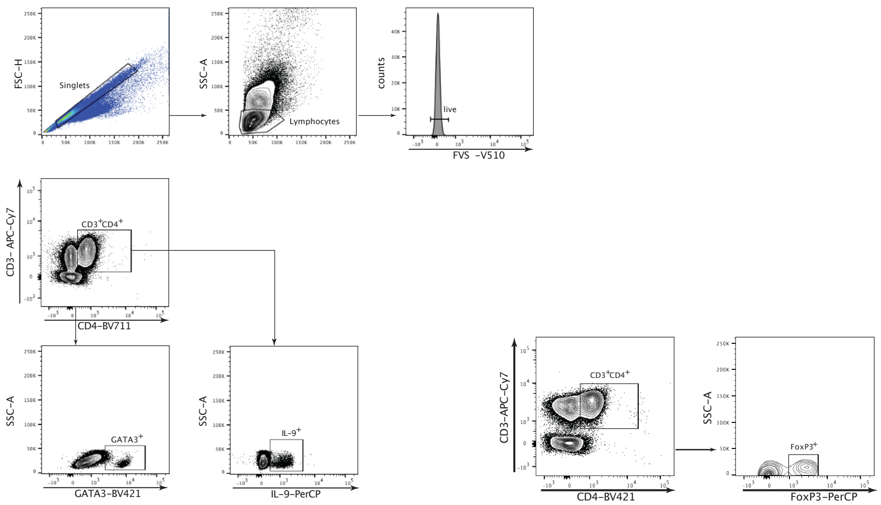

Supplement: Supplementary file 2 [file DataSheet_2.pdf]
