# Supplementary material for: TGF-β1 Drives Inflammatory Th Cell But Not Treg Cell Compartment Upon Allergen Exposure
Source: Front Immunol. 2022 Jan 7;12:763243. doi: 10.3389/fimmu.2021.763243 (PMC8777012; doi:10.3389/fimmu.2021.763243)
Supplement: Supplementary file 3 [file DataSheet_3.pdf]

Supplementary Tables

Table S1. Cytokine levels in murine BALF.

	WT +PBS (n=3-6)	WT +OVA (n=8-15)	iCD4TGFBR2 +PBS (n=3-6)	iCD4TGFBR2 +OVA (n=7-15)
IL-4 (pg/mL)	0.11±0.03***	25.0 ±5.3	0.38±0.06***	11.1±3.4##
IL-5 (pg/mL)	3.77±1.3****	54.0±8.2	2.7±0.9***	28.1±5.5#
IL-13 (pg/mL)	0.02±0.001**	10.0±2.8	0.01±0.002*	9.9±4.0
TGFβ1 (pg/mL)				
IL-9 (pg/mL)	0.2±0.1****	6.8±1.3	0.1±0.05**	3.0±1.0#
IL-6 (pg/mL)	6.0±1.6***	45.1±8.5	10.8±1.3	20.2±3.7#
IL-17A (pg/mL)	0.3±0.1*	6.4±1.3	0.3±0.06	2.2±0.9##
TNF-α (pg/mL)	11.9±5.0	21.8±2.3	14.2±4.1	10.2±1.8###
IL-1β (pg/mL)	0.3±0.1**	3.0±0.7	0.6±0.1	1.5±0.3
IFN-γ (pg/mL)	0.03±0.001**	0.2±0.07	0.03±0.002	0.1±0.06#
IL-2 (pg/mL)	0.3±0.1**	2.6±0.3	1.2±1.0	2.31±0.5
IL-10 (pg/mL)	1.7±0.8*	6.0±1.2	2.7±1.1	5.4±1.2
CXCL1	13.9±3.6*	38.5±7.2	18.0±4.7	26.4±4.4
CXCL2	10.0±2.9	15.5±3.2	10.5±4.3	11.2±1.5
CXCL10	2.3±0.5	13.9±2.3	2.9±0.9	6.3±1.2
CCL2	2.6±0.6**	13.1±2.5	8.1±2.9	8.4±1.3
CCL3	4.2±1.5*	10.3±1.6	4.4±1.6	5.7±1.3

Data are presented as mean±SEM. * indicates statistical difference compared to the respective OVA treated group. # indicates statistical difference compared to WT +OVA

Table S2. Fluorochrome-labelled antibodies in flow cytometry analyses of murine samples

Antigen	Fluorochrome	Company	Clone	Dilution
CD45	V500	BD	30-F11	1:500
CD3	A700	BioLegend	17A2	1:100
CD4	BV785	BioLegend	L3T4	1:400
CD8	E450	eBioscience	53-6.7	1:400
CD11b	BV711	BioLegend	M1/70	1:700
dTCR	PerCP-Cy5.5	BioLegend	GL3	1:300
IFNg	FITC	eBioscience	XMG1.2	1:600
FoxP3	FITC	eBioscience	FJK-16s	1:100
GATA3	PE-Cy7	eBioscience	TWAJ	1:80
RORc	PE	eBioscience	AFKJS-9	1:100
IRF4	APC	eBioscience	3EF	1:100
IL-9	PE	eBioscience	RM9A4	1:250
Zombie Aqua™ Fixable Viability Kit		BioLegend		1:1000

Table S3. Murine primers used for qPCR.

**Primer**	**Sequence**
mouse Pu.1	5'-AGAAGCTGATGGCTTGGAGC<br>5'-TTTGTCCTTGTCCACCCACC
mouse Irf4	5'-TCCTCTGGATGGCTCCAGATGG<br>5'-CACCAAAGCACAGAGTCACCTG
mouse Gata3	5'-GCTAGCCCTGACGGAGTTTTC<br>5'-AGGTGGACGTACTTTTTAACATCG
mouse Foxo1	5'-ACATTTCGTCCTCGAACCAGCTCA<br>5'-ATTTCAGACAGACTGGGCAGCGTA
mouse Foxo3	5'-CGCTGTGTGCCCTACTTC<br>5'-CCCGTGCCTTCATTCTGA
mouse Nfat5	5'-TCAGACTACCTCAACCGTTC<br>5'-TTCAGGACCAGGATCTCTTG
mouse Sgk1	5'-GGCTATCTGCACTCCCTAAACA<br>5'-CCAAAGTCAGTGAGGACGATGT
mouse Jak1	5'-ACGCTCCGAACCGAATCATC<br>5'-GTGCCAGTTGGTAAAGTAGAACC
mouse Stat6	5'-CCTGGTCGGTTCAGATGCTTT<br>5'-GTGCGGCAAGATGCTGTTTC
GAPDH	5'-CGTCCCGTAGACAAAATGGT<br>5'-TTGATGGCAACAATCTCCAC

Table S4. Patient characteristics & sputum cell differentiation (in season) of recruited patients.

	Controls (n=26)	Allergic rhinitis w/o AIT (n=9)	Allergic rhinitis with AIT (n=10)	Allergic rhinitis with asthma comorbidity w/o AIT (n=10)	Allergic rhinitis with asthma comorbidity with AIT (n=9)
**Age [years]**	27.2±1.3	28.7±2.2	26.4±1.6	26.5±1.0	26.0±0.7
**Sex (m/f)**	11/15	2/7	4/6	6/4	6/3
**GINA Score**	n.d.	n.d.	n.d.	1.1±0.4*	0.0±0.0
**mRQLQ Score**	1.2±0.2	2.5±0.3	1.3±0.4	1.1±0.4	0.8±0.4
**mRQLQ Score activity limitation**	0.1±0.1	1.3±0.3	1.0±0.3	2.0±0.3	1.0±0.2
**mRQLQ Score practical problems**	0.1±0.0	3.0±0.5	2.6±0.5	4.0±0.3	2.2±0.3
**mRQLQ Score nose symptoms**	0.2±0.1	2.8±0.5	2.3±0.5	3.6±0.3	2.6±0.3
**mRQLQ Score eye symptoms**	0.0±0.0	2.0±0.5	1.5±0.4	3.1±0.3	1.5±0.3
**mRQLQ Score other symptoms**	0.3±0.1	1.8±0.5	1.2±0.3	2.9±0.3	1.3±0.2
**IgE [IU/L]**	n.d.	147.1±48.9	40.8±14.4	147.3±100.1	110.6±77.0
**FEV1 [%]**	100.6±2.3	102.6±2.3	101.9±3.7	95.4±4.8	96.5±2.3
**FEV1 [L]**	3.7±0.2	4.2±0.3	3.9±0.1	3.8±0.2	3.5±0.2
**FVC [%]**	97.8±2.4	103.7±3.3	100.4±5.1	97.1±4.9	97.9±2.9
**FVC [L]**	4.4±0.2	4.7±0.4	4.5±0.2	4.5±0.3	4.3±0.2
**MEF25 [%]**	111.3±6.8	96.2±10.9	101.4±8.0	99.9±6.7	93.1±6.5
**MEF25 [L]**	2.2±0.1	2.2±0.4	2.4±0.2	2.1±0.2	1.8±0.3
**Total cells [$x10^4$/mL]**	47.3±8.7	134.2±31.4	42.8±9.3	178.1±94.8	19.6±2.9
**Macrophages [%]**	84.9±3.0	87.0±2.0	86.6±2.7	80.5±6.6	85.2±5.8
**Neutrophils [%]**	14.0±3.0	9.5±1.4	11.3±2.1	15.5±4.7	14.2±5.7
**Eosinophils [%]**	0.5±0.2	2.6±1.0	1.0±0.2	2.5±1.5	0.4±0.2
**Lymphocytes [%]**	0.6±0.3	0.9±0.2	1.1±0.1	2.3±1.3	0.2±0.2

Data are presented as mean±SEM. GINA score was assessed in asthma patients only; *depicts significance between allergic asthma without AIT versus allergic asthma with AIT. GINA: Global Initiative for Asthma; mRQLQ: Rhinitis quality of life mini questionnaire; IgE: Immunoglobulin E; FEV1: Forced expiratory volume in 1 second; FVC: Forced vital capacity; MEF25: Maximal expiratory flow 25%.

Table S5. Sputum cytokine levels

	Controls	Allergic rhinitis w/o AIT	Allergic rhinitis with AIT	Allergic asthma w/o AIT	Allergic asthma with AIT
**IL-4 (pg/mL)**	24.8±3.4	66.1±8.4****	14.0±2.6$^{\#\#\#}$	51.8±11.3*	14.2±2.8$^{\$\$\$}$
**IL-5 (pg/mL)**	30.8±3.9	71.2±5.9****	14.8±1.8$^{\#\#\#}$	48.8±9.7	13.6±1.7$^{\$\$\$}$
**IL-13 (pg/mL)**	9.8±1.0	19.0±2.2***	6.5±0.8$^{\#\#\#}$	14.4±2.3	5.8±0.7$^{\$\$\$\$}$
**TGFβ$_1$ (pg/mL)**	10.2±1.4	13.7±1.7	4.1±0.7$^{\#\#\#\#}$	17.0±3.1*	5.5±0.5$^{\$\$\$}$
**IL-9 (pg/mL)**	13.9±1.9	20.6±3.1	5.8±0.6$^{\#\#\#}$	19.2±4.0	4.2±0.5$^{\$\$\$}$
**IL-6 (pg/mL)**	15.5±1.7	13.2±3.3	27.1±5.0$^{\#}$	11.4±2.6	48.4±20.6$^{\$}$
**IFN-γ (pg/mL)**	6.4±0.8	4.0±0.5	9.3±1.8$^{\#\#}$	4.1±0.5	11.8±3.5$^{\$}$
**IL-2 (pg/mL)**	7.8±1.0	4.1±0.4	10.5±1.9$^{\#}$	3.2±0.3*	8.8±1.8$^{\$}$
**IL-10 (pg/mL)**	6.0±0.7	3.7±0.6	7.4±1.2$^{\#\#}$	3.3±0.3*	6.6±1.3$^{\$}$

Data are presented as mean±SEM. * indicates statistical significance compared to control subjects; # indicates statistical significance compared to untreated rhinitis patients; $ indicates statistical significance compared to untreated allergic asthma patients.

Table S6. Fluorochrome-labelled antibodies in flow cytometry analyses of human samples

Antigen	Fluorochrome	Company	Clone	Dilution
CD3	APC-Cy7	BioLegend	HIT3	1:200
CD4	BV421	BD	RPA-T4	1:200
CD4	BV711	BioLegend	OKT-04	1:200
TGFBR2	PE	R&D	25508	1:200
IL-9	eFluor710	eBioscience	MH9D1	1:100
FoxP3	PerCP-Cy5.5	eBioscience	PCH101	1:50
GATA3	BV421	BioLegend	16E10A23	1:50
FVS510	HV510	BioLegend		1:1000
