# Supplementary material for: TGF-β1 Drives Inflammatory Th Cell But Not Treg Cell Compartment Upon Allergen Exposure
Source: Front Immunol. 2022 Jan 7;12:763243. doi: 10.3389/fimmu.2021.763243 (PMC8777012; doi:10.3389/fimmu.2021.763243)
Supplement: Supplementary file 4 [file DataSheet_4.pdf]

Online supporting information

Legends for supplementary Figures.

Figure S1. Analysis of cytokines and chemokines in the soluble BALF compartment, serum, and supernatant of restimulated splenocytes.

(A) Proinflammatory cytokines and chemokines were measured in BAL fluid by chemiluminsecence assay (Mesoscale) of non-allergic WT (WT+PBS) and iCD4TGFR2 (iCD4TGFR2+PBS) and allergic WT (WT+OVA) and iCD4TGFR2 (iCD4TGFR2+OVA). Each data point represents an individual mouse. Data is compiled from one or two independent experiments (n=5-15/group). **(B)** Gating strategy to analyze the effect of OVA-induced AAI on different T cell subsets in the lung. Flow cytometric analysis of lungs was performed 24h after the last OVA-challenge of non-allergic and allergic WT and iCD4TGFR2 mice. Representative gating strategy for TH2, TH9, TH17 and Treg cells. **(C)** Levels of Il-9 were measured in the serum via ELISA. **(D-E)** Splenocyte-derived Il-9, Il-4 and Il-5 and levels of pro-inflammatory cytokines Il-2, Il-6, and TNF-α were measured by LegendPlex®. Each data point represents an individual mouse. Data is compiled from one to two independent experiments (n=5-12/group). **(F)** Flow cytometric analysis of lungs was performed 24h after the last OVA-challenge in non-allergic WT+PBS and iCD4TGFR2+PBS mice and allergic WT+OVA and iCD4TGFR2+OVA mice. Cells were pregated on single cells/live-dead/CD45+CD11b-. Each data point represents an individual mouse. Data is compiled from two independent experiments (n=6-13/group).
**p<0.01, ***p<0.001 (two-tailed Mann-Whitney U test).

Figure S2. Levels of selected cytokines in sputum of treated and untreated AR and AA patients and healthy controls out of grass-pollen season (October-January). **(A)** Levels of secreted cytokines IL-4, IL-5, IL-13, TGF-β, IL-9, IL-6, IL-2, IFN-γ and IL-10 detected in sputum supernatants of the depicted groups assessed by LEGENDplex. Data presented by individual values and mean. *$p<0.05$, **$p<0.01$, ***$p<0.001$, ****$p<0.0001$; Initially, statistical significances were assessed using a Kruskal-Wallis test and only when medians across patient groups varied significantly, multiple single comparisons were performed using two-tailed Mann-Whitney U tests. **(B)** Gating strategy for Th2, Th9, and Treg cells in peripheral blood.
